# Drug-resistant Enterobacteriaceae colonization is associated with healthcare utilization and antimicrobial use among inpatients in Pune, India

**DOI:** 10.1186/s12879-018-3390-4

**Published:** 2018-10-04

**Authors:** Renu Bharadwaj, Matthew L Robinson, Usha Balasubramanian, Vandana Kulkarni, Anju Kagal, Priyanka Raichur, Sandhya Khadse, Dileep Kadam, Chhaya Valvi, Aarti Kinikar, Savita Kanade, Nishi Suryavanshi, Ivan Marbaniang, George Nelson, Julia Johnson, Jonathan Zenilman, Jonathan Sachs, Amita Gupta, Vidya Mave

**Affiliations:** 1 Byramjee Jeejeebhoy Government Medical College-Johns Hopkins University Clinical Research Site, Pune, Maharashtra India; 2 Byramjee-Jeejeebhoy Government Medical College, Pune, Maharashtra India; 3 Johns Hopkins University School of Medicine, Baltimore, MD USA; 4 Vanderbilt University School of Medicine, Nashville, TN USA; 5 Phoenix Children’s Hospital / Maricopa Medical Center, Phoenix, AZ USA

**Keywords:** Antimicrobial resistance, Bacterial colonization, Antibiotic use, India, Prospective study

## Abstract

**Background:**

Healthcare exposure may increase drug-resistant Enterobacteriaceae colonization risk. Nascent antimicrobial stewardship efforts in low- and middle-income countries require setting-specific data. We aimed to evaluate risk factors for inpatient drug resistant Enterobacteriaceae colonization in a resource-limited setting in India.

**Methods:**

Patients age ≥ 6 months admitted with ≥24 h of fever to a tertiary hospital in Pune, India were enrolled in a prospective cohort. Perirectal swabs, collected on admission and hospitalization day 3 or 4, were cultured in vancomycin- and ceftriaxone-impregnated media to assess for ceftriaxone-resistant Enterobacteriaceae (CTRE) and carbapenem-resistant Enterobacteriaceae (CPRE). Multivariable analyses assessed risk factors for drug-resistant Enterobacteriaceae colonization among participants without admission colonization.

**Results:**

Admission perirectal swabs were collected on 897 participants; 87 (10%) had CTRE and 14 (1.6%) had CPRE colonization. Admission CTRE colonization was associated with recent healthcare contact (*p* < 0.01). Follow-up samples were collected from 620 participants, 67 (11%) had CTRE and 21 (3.4%) had CPRE colonization. Among 561 participants without enrollment CTRE colonization, 49 (9%) participants were colonized with CTRE at follow-up. Detection of CTRE colonization among participants not colonized with CTRE at admission was independently associated with empiric third generation cephalosporin treatment (adjusted odds ratio [OR] 2.9, 95% CI 1.5–5.8). Follow-up transition to CPRE colonization detection was associated with ICU admission (OR 3.0, 95% CI 1.0–8.5).

**Conclusions:**

Patients who receive empiric third generation cephalosporins and are admitted to the ICU rapidly develop detectable CTRE and CPRE colonization. Improved antimicrobial stewardship and infection control measures are urgently needed upon hospital admission.

**Electronic supplementary material:**

The online version of this article (10.1186/s12879-018-3390-4) contains supplementary material, which is available to authorized users.

## Background

Antibiotic use is increasing worldwide and has been implicated in the dramatic rise of antimicrobial resistance, which in turn threatens to reverse historical reductions in mortality for infectious diseases [1]. Few treatment options remain, for example, for Enterobacteriaceae, which are increasingly resistant to beta-lactam antibiotics due to production of extended spectrum beta-lactamase (ESBL), AmpC, and carbapenemases. Infections with drug-resistant Enterobacteriaceae, such as those producing ESBL, have been associated with increased mortality [2].

The gut serves as a reservoir for drug-resistant Enterobacteriaceae [3]. Antibiotic administration increases drug-resistant Enterobacteriaceae colonization through selection pressure and disruption of protective normal microbiota [3, 4]. Animal models show a disruption to the gut microbiome within 12 h and emergence of drug resistance genes within 3 days of antibiotic administration [5, 6]. Patients colonized with ESBL-producing Enterobacteriaceae are at greater risk for clinical infections with ESBL-producing Enterobacteriaceae than those who are not colonized [7].

In resource-limited settings, colonization with drug-resistant Enterobacteriaceae is common in adults, and, in community-based populations, associated with antibiotic use [8–10]. However, risk factors for inpatient acquisition of colonization with drug-resistant Enterobacteriaceae have not been clearly defined. As the world’s largest consumer of antibiotics [11], India has among the highest burdens of antimicrobial resistance worldwide [12–14]. Travelers from high income countries to India return home colonized with ESBL-producing Enterobacteriaceae more frequently than travelers to other countries, suggesting that India has a heavier burden of drug-resistant Enterobacteriaceae colonization compared to other low and middle income countries [15, 16].

In this study, we sought to assess the clinical factors associated with ceftriaxone- and carbapenem-resistant Enterobacteriaceae colonization among adults and children admitted to a tertiary care hospital in Pune, India. Given prior work suggesting an association with antibiotic use, we enrolled patients with acute febrile illness, a group likely to be treated with antibiotics [17].

## Methods

### Setting and participants

Between August 2013 and December 2015, we prospectively enrolled adults and children admitted to medicine and pediatric wards with acute febrile illness at Byramjee Jeejeebhoy Government Medical College – Sassoon General Hospital, Pune, India, to assess antimicrobial resistance as previously reported [18]. Sassoon General Hospital is a 1300 bed public teaching hospital in Pune, a densely populated city in Maharashtra, India with a metropolitan population exceeding 5 million. We included patients greater than 6 months of age, with self-reported or measured fever ≥38.0 °C of more than 24 h duration who were screened within 1 day of admission. We excluded inpatient transfers from other hospitals, minor-age orphans, and medical-legal cases. A dedicated study physician and social worker obtained a standardized clinical and social history at the time of enrollment. After discharge, or on day 7 of enrollment, the study physician reviewed medication administration records and laboratory investigation results from the medical record.

### Sample collection and processing

Between September 2014 and November 2015, a dedicated study nurse collected perirectal swabs on admission and on day 3 or 4 of hospitalization. Swabs were stored at − 80 °C pending processing [19, 20]. Swabs were placed into peptone broth impregnated with ceftriaxone and vancomycin. After incubating for 24 h, samples were plated onto MacConkey and sheep blood agar and incubated at 37 °C for 18 h. Isolates observed were loaded onto a Phoenix® Automated Microbiology System (Becton Dickinson) according to the manufacturer’s instructions. The Phoenix® Automated Microbiology System performs species identification and drug susceptibility testing using a cartridge-based, broth microdilution system with a redox growth indicator [21].

### Definitions

According to local hospital practice, patients < 12 years of age are considered children and admitted to the pediatric ward, and those ≥12 years of age, adolescents and adults are admitted to the medicine ward. Resistance to individual antibiotics was determined using Clinical Laboratory Standards Institute (CLSI) guidelines (2014–2015) reported by the Phoenix® system [21]. Gram-negative isolates that grew in the presence of ceftriaxone impregnated broth were considered to be ceftriaxone-resistant, with the exception of Pseudomonas isolates. ESBL- producing organisms were identified using the Phoenix® system, which employs rules to adjudicate the presence or absence of ESBL based on susceptibility to five cephalosporins alone or in combination with the beta-lactamase inhibitor, clavulanic acid [21, 22]. Enterobacteriaceae resistant to any carbapenem were considered to be carbapenem-resistant Enterobacteriaceae (CPRE) according to United States Centers for Disease Control and Prevention guidelines [23]. As defined elsewhere, drug-resistant Enterobacteriaceae colonization was defined as hospital-acquired if samples were negative at enrollment and positive at > 48 h of study [24, 25].

### Analysis

Categorical variables were assessed for individual association with initial colonization, transition to colonization with drug-resistant Enterobacteriaceae isolates, and mortality using Fisher’s exact test. The Wilcoxon rank sum test was used to assess length of hospital stay. Age was analyzed as a categorical variable stratified into five groups. A multivariable model was constructed assessing the association of predictor variables with acquisition of ceftriaxone-resistant Enterobacteriaceae (CTRE) colonization, adjusted for admission to the intensive care unit (ICU), sex, and age. Logistic regression was performed to assess predictor variables associated with all-cause mortality. Given the small sample size of CPRE colonization events, only bivariable analyses were performed. Statistical analyses were performed using R software [26].

## Results

### Study population

Between September 2014 and October 2015, 29,146 patients were admitted to adult and pediatric wards, 3589 had admission diagnoses suggestive of febrile illness, 1943 had subjective or documented fever, 1010 met all eligibility criteria and were enrolled in the parent study, 897 (89%) participants were enrolled in the substudy and had baseline perirectal swabs collected. Of the 897 study participants, 358 (40%) were children less than 12 years of age, 538 (60%) were male, 131 (15%) had been hospitalized within the past 3 months, and 214 (24%) reported using antibiotics in the month prior to admission (Table 1).

**Table 1:** Demographics and clinical characteristics of patients with and without ceftriaxone-resistant Enterobacteriaceae colonization at enrollment

Patient characteristic	Colonization with ceftriaxone-resistant Enterobacteriaceae, n (%) or median (IQR)		*p*-value
	Not colonized, *n* = 810	Colonized, *n* = 87	
Median age, years	19 (4–35)	23 (8–40)	0.07
Children (age < 12 years)	332 (41)	26 (30)	0.05
Male	487 (60)	51 (59)	0.82
Diabetes	34 (4)	5 (6)	0.42
HIV	85 (19)	11 (20)	0.86
Alcoholism	57 (7)	1 (1)	0.04
Smoking	67 (8)	7 (8)	1.00
Income < 5000 INR per month^a^	281 (35)	23 (26)	0.15
Works with animals^a^	150 (19)	20 (23)	0.31
Farmer or laborer^a^	317 (39)	32 (37)	0.73
General practitioner visit prior to hospitalization	273 (36)	37 (44)	0.19
Ayurvedic provider visit prior to hospitalization	36 (5)	8 (9)	0.12
Hospitalized within the past 3 months	113 (14)	18 (21)	0.11
Self-report of antibiotic use in the last month	188 (23)	26 (30)	0.19
Recent healthcare contact^b^	456 (56)	65 (75)	< 0.01
Admission to ICU	153 (19)	13 (15)	0.47
Cough	370 (46)	32 (37)	0.14
Diarrhea	154 (19)	16 (18)	1.00

*IQR* interquartile range, *INR* Indian rupees, ICU intensive care unit

^a^For children, this refers to the parents

^b^Composite of visit to general practitioner or ayurvedic provider, hospitalization within the past 3 months, or self-reported antibiotic use in the last month

### Drug-resistant Enterobacteriaceae colonization at admission

Of 897 participants, 91 (10%) demonstrated growth of a Gram-negative rod (GNR) in ceftriaxone-impregnated media at admission, 87 (9.7%) demonstrated growth of an Enterobacteriaceae. Colonization with CTRE at enrollment was higher in participants who had recent contact with the healthcare system including recently hospitalization, outpatient visit prior to hospitalization, or self-reported antibiotic use in the last month (Table 1). CPRE were found in 14 (1.6%) participants on admission – 10 (1.9%) adults and four (1.1%) children. An additional four (0.4%) participants grew Enterobacteriaceae with intermediate carbapenem susceptibility (Additional file 1: Table S1).

### Drug-resistant Enterobacteriaceae colonization at follow-up

**Fig. 1:**
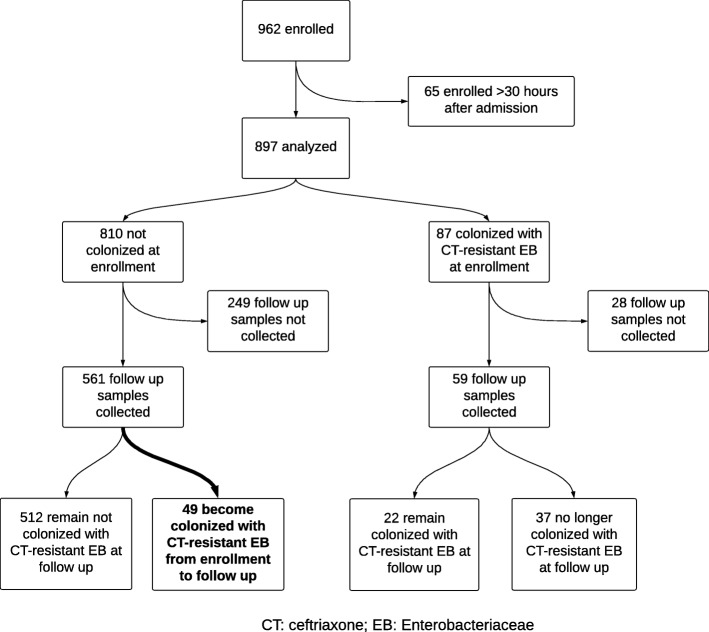
Acquisition of ceftriaxone-resistant Enterobacteriaceae colonization. Abbreviations – CTX: ceftriaxone; EB: Enterobacteriaceae

A total of 620 (69%) participants had follow-up swabs collected – 530 on Day 3 and 90 on Day 4. Of 277 (31%) participants in whom a follow-up swab could not be collected, 212 had a hospital stay of 3 days or less (157 were discharged, 31 left against medical advice, 24 died), 30 refused collection of a second sample, and for 35 a second sample could not be obtained for other reasons (Fig. 1**)**. Participants without a follow-up swab were more likely to be adults (78% vs 52%, *p* < 0.01) but were less likely to have HIV (12% vs 23%, *p* < 0.01), to have been admitted to the hospital within the past 3 months (11% vs 16%, *p* < 0.04), or to have been admitted to the intensive care unit (ICU) (13% vs 21%, *p* < 0.01). There was no difference, however, in the proportion of participants receiving antibiotics (82% vs 85%, *p* = 0.23), including third generation cephalosporins (49% vs 48%, *p* = 0.89). Of 620 participants, 78 (13%) demonstrated growth of a GNR; 67 (11%) were identified as growing CTRE. CPRE were found in 21 (3.4%) participants including 8 (2.5%) adults and 13 (4.4%) children; 2 (0.3%) participants grew Enterobacteriaceae isolates with intermediate carbapenem susceptibility.

Of 561 (90%) participants without baseline resistant Enterobacteriaceae colonization, 49 (9%) were found to have colonization at follow-up (Table 2). Extremes of age were associated with increased likelihood of CTRE colonization acquisition. Empiric therapy with a third generation cephalosporin was also associated with acquisition of CTRE colonization (71% received cephalosporin vs. 46% did not, p < 0.01) (Table 2). In multivariable analysis, adjusting for age, sex, ICU admission, and third generation cephalosporin initial antibiotic prescription, advanced age and third generation cephalosporin use remained significantly associated with acquisition of CTRE colonization, with cephalosporin use demonstrating nearly 3 times higher odds of acquisition (adjusted OR 2.9, 95% CI 1.5–5.8) (Table 2).

**Table 2:** Factors associated with detection of ceftriaxone-resistant Enterobacteriaceae colonization among 561 patients without ceftriaxone-resistant Enterobacteriaceae colonization at enrollment

Risk Factor, n (%)	Ceftriaxone-resistant Enterobacteriaceae colonization, n (%)		Unadjusted OR (95% CI)	*p*-value	Adjusted OR^a^ (95% CI)	*p*-value
	No acquisition, *N* = 512	Acquisition, *N* = 49				
Age in years						
< 5	163 (32)	21 (43)	4.7 (1.3–29.9)	0.04	4.4 (1.2–28.7)	0.06
5–11	88 (17)	5 (10)	2.1 (0.4–14.8)	0.39	2.0 (0.4–14.6)	0.41
12–23	73 (14)	2 (4)	Ref	Ref	Ref	Ref
24–59	154 (30)	15 (31)	3.6 (1–22.9)	0.1	3.8 (1–24.9)	0.08
≥ 60	34 (7)	6 (12)	6.4 (1.4–45.5)	0.03	7.0 (1.5–50.1)	0.02
Male sex	307 (60)	33 (67)	1.4 (0.7–2.8)	0.36	1.5 (0.8–2.9)	0.23
Income < 5000 INR / month	183 (36)	23 (47)	1.6 (0.0–8-3)	0.12		
Farmer or laborer	203 (40)	24 (49)	1.5 (0.8–2.7)	0.22		
HIV^b^	56 (22)	6 (27)	1.3 (0.4–3.7)	0.6		
ICU	102 (20)	17 (35)	2.1 (1.1–4.1)	0.03	1.6 (0.8–3.4)	0.18
Pre-hospital antibiotic use						
Within the past week	99 (19)	13 (27)	1.5 (0.7–3.0)	0.26		
Within the past month	121 (24)	14 (29)	1.3 (0.6–2.6)	0.48		
Initial inpatient antibiotic Rx						
Any antibiotic	432 (84)	45 (92)	2.1 (0.7–8.2)	0.21		
Multiple antibiotics	240 (47)	30 (61)	1.8 (0.9–3.5)	0.07		
3rd-gen cephalosporin	234 (46)	35 (71)	3.0 (1.5–6.1)	< .01	2.9 (1.5–5.8)	< 0.01
Fluoroquinolone	19 (4)	1 (2)	0.5 (0.0–3.6)	1		
Macrolide	96 (19)	13 (27)	1.6 (0.7–3.2)	0.19		
Aminoglycoside	53 (10)	8 (16)	1.7 (0.6–3.9)	0.23		

*OR* odds ratio, *IQR* interquartile range, *ICU* intensive care unit, *INR* Indian rupees, *Rx* prescription

^a^Adjusted for age, sex, ICU admission, and third generation cephalosporin initial antibiotic prescription

^b^HIV test results were available for 249 patients without ceftriaxone-resistant Enterobacteriaceae acquisition and 22 patients with ceftriaxone-resistant Enterobacteriaceae acquisition

There were 19 (3%) participants (13 adults and 6 children) who on admission were not colonized with CPRE, but were found to have CPRE colonization on follow-up. Children under age 5 were most likely to acquire CPRE colonization compared to other age categories (OR 4.1, 95% CI 1.3–18.4). Participants who acquired CPRE were more likely to be admitted to the ICU (OR 3.0, 95% CI 1.0–8.5) and more likely to have received empiric aminoglycosides (OR 4.2, 95% CI 1.2–4.3). There was notably an association between ICU admission and aminoglycoside use, *p* < 0.001.

Among 59 (9%) participants found to have CTRE colonization at enrollment in whom follow-up samples were collected, colonization remained detectable in 22 (37%) participants (Fig. 1). CTRE Use of third-generation cephalosporins was not associated with loss of detection of CTRE colonization (*p* = 0.79). Among the 14 participants found to have CPRE colonization at enrollment, follow-up samples were collected on 12 participants, of which two (17%) continued to demonstrate CPRE colonization.

### Perirectal isolate species and antimicrobial resistance patterns

The 136 participants with CTRE identified in either enrollment or follow-up samples comprised 52 (14%) of the enrolled children and 84 (16%) of the enrolled adults. Five specimens grew two different CTRE species. Of the 101 ceftriaxone-resistant *Escherichia coli* isolates, 77 (76%) were identified as ESBL producers by Phoenix®, while 32 of 49 (65%) *Klebsiella* isolates were identified as ESBL producers (Table 3). Though the Phoenix® system does not report the presence of AmpC as part of the resistance profile for identified organisms, cefoxitin resistance may suggest the presence of AmpC, particularly in the context of a ceftriaxone-resistant organism that is not flagged as an ESBL organism by Phoenix®. Both *E. coli* and *Klebsiella* species were mostly resistant to cefoxitin – 66 and 63% respectively. None of the 11 *Enterobacter* isolates were considered to be ESBL isolates by Phoenix®, and all were resistant to cefoxitin, suggesting that AmpC was the likely mechanism of resistance for the *Enterobacter* isolates.

**Table 3:** Isolate species and resistance pattern among 179 Gram-negative perirectal isolates

Species	n (%)	ESBL, n (%)	Cefoxitin resistant, n (%)	Carbapenem resistant, n (%)
All isolates	179 (100)	–	–	38 (21)
*Pseudomonas* species	14 (8)	–	–	2 (14)
*Comamonas* species	2 (1)	–	–	0
*Moraxella* species	1 (0.6)	–	–	0
All Enterobacteriaceae	162 (91)	109 (67)	110 (68)	36 (22)
*Escherichia coli*	101 (78)	77 (76)	67 (66)	16 (15)
*Klebsiella pneumoniae*	49 (27)	32 (65)	31 (63)	16 (33)
*Enterobacter* species	11 (6)	0	11 (100)	3 (27)
*Citrobacter farmeri*	1 (0.6)	0	1 (100)	1 (100)

*ESBL* extended spectrum beta-lactamase

### Clinical outcomes

The median length of stay among the 897 participants was 4.0 days, interquartile range (IQR) (3–7); 4.0 days (IQR 2–6) for adults and 5.5 days (IQR 4–9) for children. Among participants who had follow-up samples collected, median length of stay was 6.0 days (IQR 4–9). There was no association with length of stay and Enterobacteriaceae colonization at follow-up, *p* = 0.35. There were 34 deaths (6%) among participants with collected follow-up samples. Participants who were colonized at follow-up were more likely to die. Among 71 participants who were colonized with CTRE at follow-up there were 8 (11%) deaths, whereas among 549 participants who were not colonized there were 26 (5%) deaths (*p* = 0.02) (Table 4). In a multivariable model adjusted for age <  12 years and admission to the ICU, CTRE colonization was no longer associated with mortality (AOR 2.4, 95% CI 0.9–6.0) (Table 4). There were three (9%) deaths among participants who were colonized with CPRE at enrollment or follow-up.

**Table 4:** Factors associated with mortality among patients who completed follow-up (*N* = 580)^a^

Clinical Factor	Survived, *N* = 546, n (%)	Died, *N* = 34, n (%)	Unadjusted OR (95% CI)	*p*-value	Adjusted OR^b^ (95% CI)	*p*-value
Male sex	321 (59)	23 (68)	1.5 (0.7–3.4)	0.37	–	–
Child < 12 years	267 (49)	11 (32)	0.5 (0.2–1.1)	0.08	0.2 (0.1–0.4)	< 0.01
Income < 5000 INR / month	197 (36)	15 (44)	1.4 (0.6–3)	0.36	–	–
Diabetes	18 (3)	1 (3)	0.9 (0–6)	1	–	–
HIV	56 (10)	7 (21)	2 (0.6–5.7)	0.17	–	–
Diarrhea	110 (20)	9 (26)	1.4 (0.6–3.3)	0.38	–	–
Cough	278 (51)	13 (38)	0.6 (0.3–1.3)	0.16	–	–
ICU admission	96 (18)	19 (56)	6.8 (3.1–15.6)	< .01	14.7 (6.1–36.9)	< 0.01
Admission ceftriaxone-resistant EB colonization	52 (10)	2 (6)	0.6 (0.1–2.4)	0.76	–	–
Follow-up ceftriaxone-resistant EB colonization	53 (10)	8 (24)	2.9 (1.1–6.9)	0.02	2.4 (0.9–6.0)	0.06
Acquisition of ceftriaxone-resistant EB colonization	37 (7)	6 (18)	2.8 (0.9–7.7)	0.04	–	–

*OR* odds ratio, *INR* Indian Rupees, *ICU* intensive care unit, *EB* Enterobacteriaceae

^a^Mortality data was not available for 40 (6%) of patients who completed follow-up perirectal swab collection

^b^Adjusted for child < 12 years, ICU admission, and colonization with ceftriaxone-resistant EB at follow-up

## Discussion

Our study has several key findings. First, we identified a 10% prevalence of community-acquired ceftriaxone-resistant GNR colonization and 1.6% colonization with CPRE among Indian adults and children admitted with acute febrile illness. Second, we observed that healthcare contact was associated with increased odds of admission CTRE colonization. Third, we found that participants without baseline CTRE colonization who received empiric third generation cephalosporins had almost three-fold higher odds of follow up detection of CTRE colonization, and were more likely to acquire CPRE colonization if admitted to the ICU.

The 10% rate of community-acquired CTRE colonization reported in this study is comparable to rates of 10–15% reported in studies that used rectal and perirectal swabs to detect colonization [27, 28], and to another study of children in India showing 13% of children to have ceftriaxone-resistant *E. coli* colonization [29]. The rate was lower than the 23–69% ESBL-Enterobacteriaceae colonization rate reported among healthy adults from other resource limited Asian settings that determined colonization using higher inoculum stool samples [8, 9]. While obtaining a stool specimen from healthy volunteers at a time of convenience may be practical for community surveillance studies, in order to assess dynamic colonization during hospitalization, perirectal swab collection at specific time intervals is more practical than stool culture.

Admission CTRE colonization was no longer detected at follow-up for almost two-thirds of CTRE colonized participants, a higher than expected proportion. Although some studies have shown persistence of multidrug-resistant Enterobacteriaceae for months after acquisition, others have shown that with daily screening, more than half of patients demonstrate intermittent colonization [25, 30, 31]. As we only performed one follow-up culture, it is possible that further follow-up cultures may have revealed persistence of colonization in additional participants. However, because third-generation cephalosporin use was not associated with loss of detectable ceftriaxone-resistant GNR colonization, limitations in the CTRE colonization detection modality were not impacted by third-generation cephalosporin use.

Use of empiric third generation cephalosporins was associated with detection of CTRE colonization among participants not found to be colonized at admission, even when adjusted for other factors. Empiric aminoglycosides use was associated with acquisition of carbapenem-resistant Enterobacteriaceae colonization, but was likely confounded by ICU admission. From this study, we cannot determine if follow-up detection of drug-resistant Enterobacteriaceae colonization among participants without baseline colonization was due to nosocomial transmission or overgrowth of minority drug-resistant colonies in the presence of selection pressure from administered antibiotics. Most nosocomial acquisition of drug-resistant Enterobacteriaceae in studies conducted in resource-rich settings is not attributed to transmission from other hospitalized patients [32, 33]. However, a study in a resource-limited setting showed genetic similarities among acquired isolates suggesting nosocomial cross-transmission [34].

Previous work in a resource-rich ICU setting has shown an association between drug-resistant Enterobacteriaceae colonization and mortality [35], but similar data from resource-limited settings describing clinical associations with colonization is limited. We found that participants with CTRE colonization at follow-up were more likely to die in an unadjusted analysis. After adjusting for ICU admission and age, the association no longer remained statistically significant. Any association of CTRE colonization and mortality does not necessarily signify causality, as there may be unmeasured risk factors common to both CTRE colonization acquisition and mortality.

Unfettered antibiotic use has been implicated as one of the key factors driving global increasing antimicrobial resistance [1]. Studies in other settings have demonstrated that antibiotic administration and ICU admission are associated with development of antibiotic-resistant bacterial stool colonization during the course of a hospitalization, but used longer intervals between enrollment and follow-up [28, 36]. The finding that initial empiric antibiotic choice can also significantly impact this process is concerning, especially given how commonly third generation cephalosporins are used in India and elsewhere.

Antimicrobial use may be tempered by antimicrobial stewardship policies, which were not yet in place at the time of this study. Guidelines offer two general antimicrobial stewardship approaches: preauthorization of antibiotics and auditing of antibiotic prescriptions after treatment initiation [37, 38]. De-escalation of antibiotic therapy was associated in a meta-analysis with decreased mortality risk [39]. However, it remains unclear how quickly antibiotics should be de-escalated. Rapid acquisition of detectable resistant Enterobacteriaceae colonization, as noted in our study, may result in a narrow window of opportunity for prevention of drug-resistant organism colonization. A recent study notably failed to show a reduction in the emergence of multidrug-resistant Gram-negative infections after de-escalation of anti-pseudomonal beta-lactams [40]. Our study design required participants to remain hospitalized through Day 3 or Day 4, which excluded both those with early mortality, and those with mild illness who were quickly discharged. However, among patients who do remain hospitalized through the third or fourth day of admission, the contribution of empiric antibiotics to drug-resistant Enterobacteriaceae colonization poses an antimicrobial stewardship concern.

## Conclusions

In conclusion, colonization with drug-resistant Enterobacteriaceae is common among patients admitted with fever in Pune, India. Our study highlights the need for improved antimicrobial stewardship and infection control measures, which the World Health Organization acknowledges in its Global Action Plan on Antimicrobial Resistance [41]. Physicians and governing bodies in India have recognized the threat of antimicrobial resistance responding with newly introduced legislation which requires a prescription for the sale of many advanced antibiotics including third and fourth generation cephalosporins [12, 42]. Continued surveillance of drug-resistant Enterobacteriaceae colonization in India and other resource-limited settings is warranted.

## Additional file

Additional file 1: **Table S1.** Demographics and clinical characteristics of patients with and without carbapenem-resistant Enterobacteriaceae colonization at enrollment. (PDF 43 kb)

## Abbreviations

CPRE: Carbapenem-resistant Enterobacteriaceae
CTRE: Ceftriaxone-resistant Enterobacteriaceae
EB: Enterobacteriaceae
ESBL: Extended spectrum beta-lactamase
GNR: Gram-negative rod
ICU: Intensive care unit
INR: Indian rupees
IQR: Interquartile range
OR: Odds ratio
Rx: Prescription
