# Supplementary material for: Drug-resistant Enterobacteriaceae colonization is associated with healthcare utilization and antimicrobial use among inpatients in Pune, India
Source: BMC Infect Dis. 2018 Oct 4;18:504. doi: 10.1186/s12879-018-3390-4 (PMC6172743; doi:10.1186/s12879-018-3390-4)
Supplement: Supplementary file 1 — Table S1. Demographics and clinical characteristics of patients with and without carbapenem-resistant Enterobacteriaceae colonization at enrollment. (PDF 43 kb) [file 12879_2018_3390_MOESM1_ESM.pdf]

Supplementary table: Demographics and clinical characteristics of patients with and without carbapenem-resistant Enterobacteriaceae colonization at enrollment			
Patient characteristic	Not colonized, N=883, n (%) or median (IQR)	Colonized, N=14, n (%) or median (IQR)	p-value
Age	20 (4-35)	30 (8-39.5)	0.36
Male	487 (55)	7 (50)	0.58
Diabetes	34 (4)	1 (7)	0.47
HIV*	85 (17)	4 (40)	0.1
Vegetarian	89 (10)	4 (29)	0.06
Alcoholism	57 (6)	0 (0)	-
Smoking	67 (8)	1 (7)	1
Income < 5,000 INR per month	281 (32)	2 (14)	0.16
Works with animals	150 (17)	3 (21)	0.74
Farmer or laborer	317 (36)	5 (36)	1
General practitioner visit prior to hospitalization	273 (33)	4 (31)	0.78
Ayurvedic provider visit prior to hospitalization	36 (4)	1 (8)	0.51
Hospitalized within the past 3 months	113 (13)	6 (43)	<0.01
Surgery within the past 3 months	9 (1)	0 (0)	-
Self-report of antibiotic use in the last month	188 (21)	5 (36)	0.34
Admission to ICU	153 (17)	3 (21)	0.73
Cough	370 (42)	6 (43)	1
Diarrhea	154 (17)	5 (36)	0.16

IQR: interquartile range; INR: Indian Rupees; ICU: intensive care unit

*HIV test results were available for 497 patients without enrollment carbapenem-resistant Enterobacteriaceae colonization and 10 patients with enrollment carbapenem-resistant Enterobacteriaceae colonization
